# Bidirectional associations between influenza and COVID-19 vaccination: a systematic review and meta-analysis

**DOI:** 10.3389/fpubh.2026.1756985

**Published:** 2026-06-17

**Authors:** Xu Luo, Jing-Shan Deng, Jia-Ni Chen, Ting Ye, Si-Jia Chen, Huang-Lin Su, Tao-Hsin Tung, Jian-Sheng Zhu

**Affiliations:** 1 Department of Infectious Diseases, Taizhou Hospital of Zhejiang Province Affiliated to Wenzhou Medical University, Linhai, Zhejiang, China; 2 Evidence-based Medicine Center, Taizhou Hospital of Zhejiang Province Affiliated to Wenzhou Medical University, Linhai, Zhejiang, China

**Keywords:** COVID-19 vaccine, influenza vaccine, meta-analysis, vaccination history, vaccine willingness, vaccination behavior

## Abstract

**Purpose:**

This study aimed to investigate the effect of vaccination history on future willingness or behavior to receive vaccinations for influenza and COVID-19.

**Methods:**

In this systematic review and meta-analysis, the PubMed, Embase, Web of Science, Cochrane Library, APA PsycInfo, CINAHL and Scopus databases were searched up to February 28, 2026, to identify relevant studies in accordance with the inclusion and exclusion criteria. The analysis ultimately included 145 population-based studies conducted in the United States, China, Thailand, the United Arab Emirates, Egypt, and other countries.

**Results:**

Prior vaccination histories for influenza and COVID-19 interacted with each other and were associated with an increased willingness and likelihood to receive future vaccines. Specifically, having received the influenza vaccine was associated with an increased willingness to receive the COVID-19 vaccine(odds ratio [OR] = 2.73, 95% confidence interval [CI]: 2.34–3.19, 95% prediction interval:[PI] 0.66–11.07), as well as with an increased likelihood of actually receiving the COVID-19 vaccine (OR = 2.76, 95% CI: 2.32–3.29, 95%PI: 0.90–8.52). Receiving a COVID-19 vaccine was associated with an increased willingness to receive a influenza vaccine (OR = 3.04, 95% CI: 1.25–7.39), and was also associated with an increased likelihood of actually receiving the influenza vaccine (OR = 3.91, 95% CI: 2.45–6.24, 95%PI: 0.81–18.81).

**Conclusion:**

In conclusion, prior vaccination history was associated with higher odds of future vaccination, and influenza and COVID-19 vaccination experiences influenced each other. Influenza and COVID-19 vaccine promotion strategies can likely be combined to increase overall vaccination rates.

**Systematic review registration:**

PROSPERO identifier CRD42024594174.

## Introduction

Seasonal influenza and COVID-19 are major public health threats that seriously impact population health (1, 2) and cause significant morbidity and mortality (3, 4). Since 2020, seasonal influenza and COVID-19 have circulated simultaneously during specific periods (5, 6). Influenza viruses and severe acute respiratory syndrome coronavirus 2 (SARS-CoV-2) are similar (7); they both cause infectious respiratory illnesses with similar symptoms, and similar populations are at risk for both diseases (5). Additionally, mixed infections with SARS-CoV-2 and influenza are associated with higher risks of severe illness and death (8, 9); therefore, the combination of influenza and COVID-19 vaccines has been promoted as an effective public health strategy (10–12).

Influenza and COVID-19 vaccination behaviors affect each other. A study of healthcare workers revealed that those willing to receive influenza vaccination were more willing to receive the COVID-19 vaccine (13). Additionally, a study conducted in Poland revealed that those who received the influenza vaccine were more willing to receive COVID-19 vaccine boosters (14). A Greek study reported similar findings: those who received a booster shot of the COVID-19 vaccine were more likely to receive the influenza vaccine (15). Another study conducted in Colombia revealed that those who planned to receive the influenza vaccine were also more likely to receive the COVID-19 vaccine (16).

Despite the importance of vaccines in protecting the health of the population, vaccine hesitancy—i.e., the delay in acceptance or the refusal of vaccines despite their availability—remains prevalent (17). Vaccine hesitancy affects vaccination coverage and increases infection and mortality rates (18–20). If the relationship between influenza and COVID-19 vaccination can be clarified, resources can be pooled to promote vaccination in a targeted and strategic manner (21). This study aimed to explore the relationship between influenza and COVID-19 vaccination to promote vaccination and protect public health.

## Methods

### Literature search

We searched the PubMed, Embase, Web of Science, Cochrane Library, APA PsycInfo, CINAHL and Scopus databases up to February 28, 2026. The search was conducted using key terms such as COVID-19 vaccine and influenza vaccine; Medical Subject Heading terms; and combination of Boolean logic operators (“AND,” “OR,” and “NOT”). The study strategy is shown in Supplementary material 1. We also identified additional relevant articles by reviewing the reference lists of the selected studies. This study was conducted in accordance with the Preferred Reporting Items for Systematic Reviews and Meta-Analyses (PRISMA) guidelines. The protocol for this study has been registered in PROSPERO with the identifier CRD42024594174.

### Study selection

All the documents were selected based on the Population, Exposure, Comparator, Outcomes and Study design (PECOS) framework, as follows: (a) Participants: studies reporting vaccination status or willingness of individuals themselves. Studies in which vaccination decisions were made by parents or guardians on behalf of children were excluded; (b) Exposure: history of influenza vaccination or COVID-19 vaccination; (c) Comparator: people with no history of influenza vaccine or COVID-19 vaccination; (d) Outcomes: odds ratios (ORs) indicating the effect of influenza vaccine/COVID-19 vaccination history on the willingness to receive vaccines or vaccination behavior (uptake) in the future (COVID-19 vaccine/influenza vaccine); (e) Study design: cross-sectional, cohort and case–control studies. We excluded (a) unpublished articles; (b) review articles; and (c) articles published in the form of conference abstracts, comments, or letters.

After deleting duplicates, the titles and abstracts of the retrieved studies were screened, and the full texts of the studies that met the inclusion criteria were retrieved. We comprehensively analyzed the full texts of the included studies to identify potentially relevant information. LX and DJS independently performed duplicate screenings of the titles, abstracts and full texts, and any disagreements were resolved by a third investigator (ZJS). The agreement rates between the reviewers during title and abstract screening, full-text screening were 0.80 and 0.70.

### Data extraction and quality evaluation

Data were extracted and summarized in a predefined data extraction form in Microsoft Excel software. Data extraction was carried out independently by two investigators (LX and DJS), and any discrepancies were resolved by a third investigator (ZJS). The following data were extracted: study characteristics (i.e., first author’s name, year of publication, country, and type of study), population characteristics (i.e., sample size, age, status), adjusted confounders, and outcome indicators. Our primary outcome of interest was the odds ratio (OR) for future vaccination intention or vaccination behavior (uptake) between previously vaccinated and unvaccinated populations, with estimates calculated using 95% confidence intervals (CIs).

The quality of the cross-sectional studies was evaluated by using the Joanna Briggs Institute (JBI) tool (22). Case–control studies and cohort studies were assessed for quality using the NOS scale (23). Specific evaluation items for the JBI scale and NOS scale are shown in Supplementary material 2. The level of agreement between the reviewers in the quality assessment was 0.70.

### Statistical analysis

The meta-analysis was conducted using Stata software 18.0 (StataCorp, College Station, TX, USA). Odds ratios (ORs) and their corresponding 95% confidence intervals (CIs) were calculated for each included study. A random-effects meta-analysis model was applied using the DerSimonian-Laird (D-L) estimator for the between-study variance (τ^2^), which is also used to quantify heterogeneity via the *I*^2^ statistic.

To explore potential sources of heterogeneity, we conducted pre-specified subgroup analyses based on *a priori* hypotheses, including study population and study country. Formal tests for subgroup differences were performed using Cochran’s Q test for subgroup interactions; a *p*-value < 0.05 for subgroup differences was considered statistically significant. In cases where substantial heterogeneity was detected, we further conducted sensitivity analyses by sequentially omitting each study to assess the robustness of the pooled estimates, as well as meta-regression to examine the potential influence of continuous moderators, including study year and sample size. A *p*-value < 0.05 was considered statistically significant for meta-regression coefficients. Publication bias was evaluated using funnel plots and Egger’s test in Stata software 18.0.

## Results

### Characteristics of the studies included

**Figure 1:**
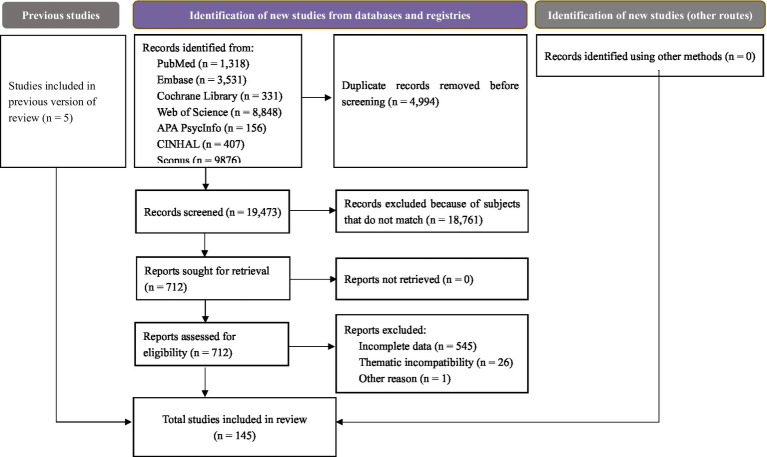
PRISMA flow chart.

A comprehensive search of the databases yielded 24,467 articles; after removing duplicates, 19,473 articles remained. Subsequently, 18,761 articles were excluded after screening their titles and abstracts. After the full texts of the remaining 712 articles were screened, 140 were eligible for inclusion. Additionally, five articles were identified from the reference lists of the eligible studies. Therefore, 145 articles published between 2020 and 2026 were ultimately included in this study. The article screening process is shown in Figure 1. The included studies comprised cross-sectional studies, and cohort studies, with sample sizes ranging from 55 to 3,344,855 participants. These studies were conducted in the United States, China, Egypt, Thailand, the United Arab Emirates, and other locations. Due to its length, the table of data characteristics for the included studies is presented in Supplementary material 3.

Table 1 presents cohort studies evaluated using the NOS scale. Cross-sectional studies were evaluated using the JBI scale; however, due to the large number of included studies and the resulting length of the text, the evaluation of cross-sectional studies is provided in Supplementary material 4. The majority of included cross-sectional studies demonstrated adequate methodological quality, with JBI scores exceeding 70% in most domains. Similarly, most cohort studies received Newcastle-Ottawa Scale scores above 7, indicating generally good quality. However, it is important to note that all included studies were observational in nature; therefore, some degree of residual confounding and inherent bias cannot be excluded. The quality assessment results should be interpreted with caution.

**Table 1:** Quality evaluation based on the NOS.

Cohort studies									
Study	Selection				Comparability	Outcome			Quality score
	Representativeness of the exposed cohort	Selection of the non-exposed cohort	Ascertainment of exposure	Demonstration that outcome of interest was not present at start of study	Comparability of cohorts on the basis of the design or analysis	Assessment of outcome	Was follow-up long enough for outcomes to occur	Adequacy of follow-up of cohorts	
Ha et al.	1	0	1	1	2	1	1	1	8
Qureshi et al	1	1	1	1	2	1	1	1	9
Shariff et al	1	1	1	1	2	1	1	1	9
Giles et al.	1	1	1	1	2	1	1	0	8
Groom et al.	1	0	1	1	1	1	1	1	7

### Influence of influenza vaccination history on willingness to receive the COVID-19 vaccination

Among the studies included, 91 studies found that a history of influenza vaccination was associated with a higher willingness to receive the COVID-19 vaccine (24–113). These studies were published between 2020 and 2025, with sample sizes ranging from 55 to 32,361. The study populations included the general public, healthcare workers, and specific groups (such as older adults, pregnant women, and people with chronic diseases). A meta-analysis using a random-effects model of these studies showed that a history of influenza vaccination was associated with increased willingness to receive the COVID-19 vaccine (OR = 2.73, 95% CI: 2.34–3.19). The forest map is quite long; please refer to Supplementary material 5 for a clear and complete version. Due to the high heterogeneity among the included studies, the 95% confidence interval for the pooled odds ratio ranged from 0.66 to 11.07, suggesting that the true effect size may vary across different study settings. Therefore, this pooled odds ratio (OR = 2.73, 95% CI: 2.34–3.19) can be interpreted as the average association across different populations and settings and cannot be directly extrapolated to any specific scenario.

Due to the high heterogeneity among the included studies, a subgroup analysis was conducted based on the study country and study population. We conducted a subgroup analysis for countries with 5 or more studies; the results are shown in Table 2. After grouping, the heterogeneity of the studies from China, Italy, and Saudi Arabia decreased to 63.3, 86.8, and 78.6%, respectively. In addition, subgroup analyses were conducted for different study populations; the results are shown in Table 3. There was a slight reduction in heterogeneity among studies involving the general population and healthcare workers. Of note, the Q-tests revealed significant subgroup differences in both subgroup analyses by study country and by study population, suggesting that study country and study population may be contributing factors to the high heterogeneity observed across the included studies. We further conducted sensitivity analyses and meta-regression on the included studies. After applying the leave-one-out method, the range of the pooled OR values stabilized between 2.65 and 2.78, and no single study was found to have a dominant influence on the overall results. Meta-regression analyses, with study year (*p* = 0.690) and sample size (*p* = 0.246) as covariates, found no significant associations; thus, the high heterogeneity among the included studies is likely not attributable to time trends or sample size.

**Table 2:** Subgroup analysis by country (influenza vaccination history and COVID-19 vaccination willingness).

Subgroup	Number of studies	Pooled OR	95% CI	Heterogeneity I^2^ (%)	*p*-value for within-subgroup heterogeneity
China	12	1.78	(1.53, 2.08)	63.3	0.002
Saudi Arabia	6	1.80	(1.42, 2.27)	78.6	<0.001
United States	12	3.55	(2.16, 5.83)	96.6	<0.001
Italy	8	2.62	(1.73, 3.95)	86.8	<0.001

Test for subgroup differences: Q_between = 9.27, df = 3, *p* = 0.026. *p*-value for within-subgroup heterogeneity from Q-test. All subgroups were analyzed using the DerSimonian-Laird random-effects model.

**Table 3:** Subgroup analysis by study populations (influenza vaccination history and COVID-19 vaccination willingness).

Subgroup	Number of studies	Pooled OR	95% CI	Heterogeneity *I*^2^ (%)	*p*-value for within-subgroup heterogeneity
General populations	29	2.09	(1.80, 2.42)	93.7	<0.001
Specific populations	30	3.15	(2.11, 4.72)	98.6	<0.001
Healthcare workers	34	2.97	(2.50, 3.53)	89.1	<0.001

Test for subgroup differences: Q_between = 10.61, df = 1, *p* = 0.005. *p*-value for within-subgroup heterogeneity from Q-test. All subgroups were analyzed using the DerSimonian-Laird random-effects model.

**Figure 2:**
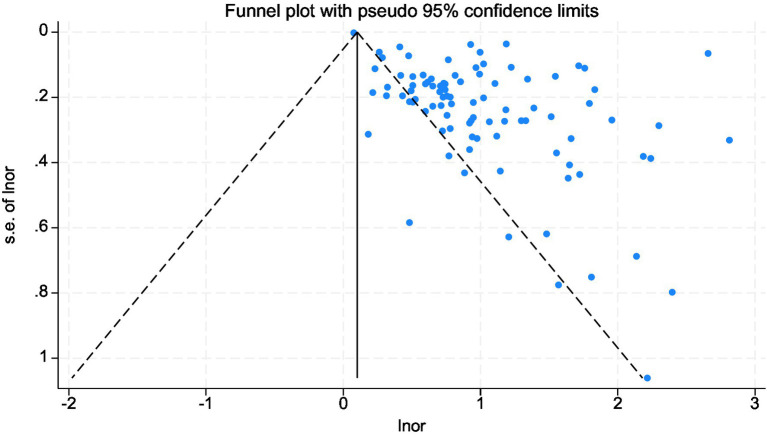
Funnel plot of the included studies (influence of influenza vaccination history on willingness to receive the COVID-19 vaccine).

The funnel plot for this analysis shows slight asymmetry (see Figure 2); the Egger test confirmed the presence of bias (*t* = 5.22, *p* < 0.001), suggesting that the small-sample studies included in the analysis may have overestimated the effect size. However, the trim-and-fill method did not identify any studies requiring imputation (number of imputations = 0), and the adjusted OR was identical to the original estimate. Therefore, we continue to report the unadjusted pooled effect size and acknowledge the potential impact of publication bias in the discussion.

### Impact of influenza vaccination history on COVID-19 vaccination behavior

**Figure 3:**
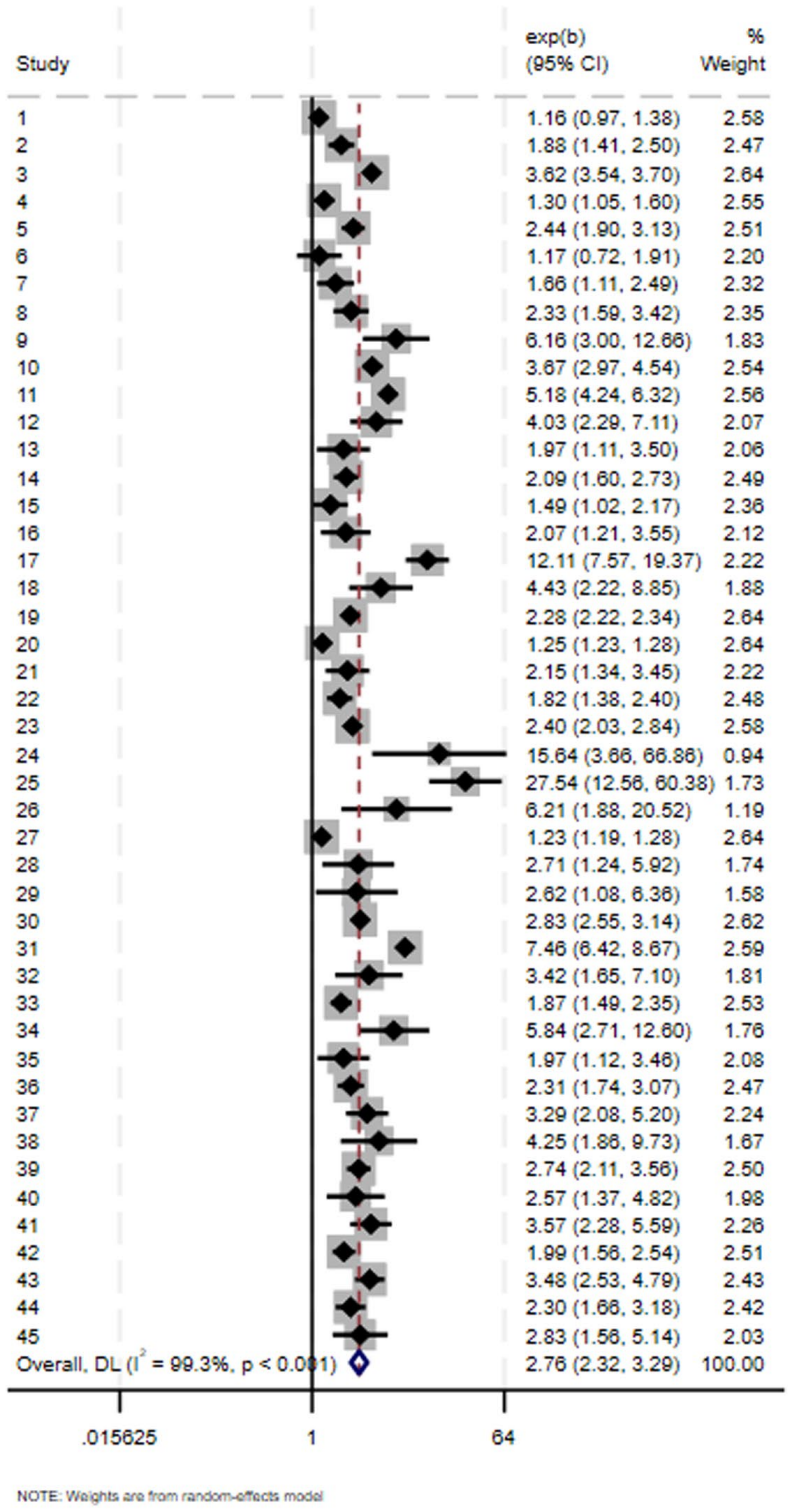
Odds ratio of influenza vaccination history with COVID-19 vaccination behavior (yes vs. no).

Among the 44 studies included in the analysis, it was found that a history of influenza vaccination was associated with COVID-19 vaccination behavior (4, 21, 26, 95, 114–153). These studies were published between 2021 and 2026, with sample sizes ranging from 196 to 3,474,874. The study populations included the general public, healthcare workers, and specific groups (such as older adults, pregnant women, patients with chronic diseases, and people living with HIV). A meta-analysis of random-effects models showed that a history of influenza vaccination was associated with higher COVID-19 vaccination rates in the population (OR = 2.76, 95% CI: 2.32–3.29), see Figure 3 for the forest plot. There was also a high degree of heterogeneity among the included studies (*I*^2^ = 99.3%, *p* < 0.001). The 95% confidence interval for the OR was 0.90 to 8.52; this pooled OR should be interpreted as the average association across different populations and settings.

**Figure 4:**
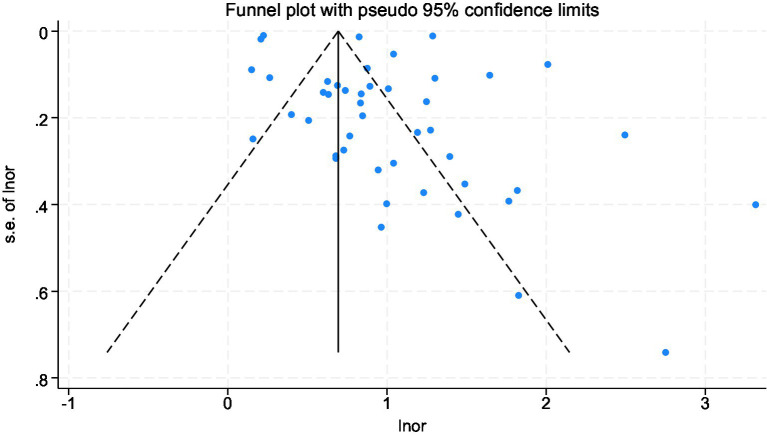
Funnel plot of the included studies (influence of influenza vaccination history on COVID-19 vaccination behavior).

To address the high level of heterogeneity, the included studies will be analyzed by subgroup based on the country of study and the study population. A subgroup analysis was conducted for countries with 5 or more studies; the results are shown in Table 4, but heterogeneity remained high across all groups. Subgroup analysis by study population revealed a significant reduction in heterogeneity in the healthcare workers subgroup (*I*^2^ = 53.0%), but heterogeneity remained high in the subgroups comprising the general population and specific populations (see Table 5). The between-subgroup Q-tests were not significant, suggesting that study country and study population may not be the main sources of heterogeneity. To address the high heterogeneity, we conducted sensitivity analyses and meta-regression on this subset of studies. After applying the leave-one-out method, the pooled OR range remained stable at 2.22–3.44, and no single study was found to dominate the overall results. Meta-regression analyses using study year (*p* = 0.277) and sample size (*p* = 0.708) as covariates did not reveal any significant associations. The Egger test indicated no significant publication bias (*t* = 1.91, *p* = 0.361), and the funnel plot was largely symmetrical (Figure 4).

**Table 4:** Subgroup analysis by country (influenza vaccination history and COVID-19 vaccination behavior).

Subgroup	Number of studies	Pooled OR	95% CI	Heterogeneity *I*^2^ (%)	*p*-value for within-subgroup heterogeneity
Greece	6	2.52	(1.76, 3.63)	86.8	<0.001
United States	13	2.80	(2.37, 3.31)	89.1	<0.001

Test for subgroup differences: Q_between = 0.26, df = 1, *p* = 0.610. *p*-value for within-subgroup heterogeneity from Q-test. All subgroups were analyzed using the DerSimonian-Laird random-effects model.

**Table 5:** Subgroup analysis by study populations (influenza vaccination history and COVID-19 vaccination behavior).

Subgroup	Number of studies	Pooled OR	95% CI	Heterogeneity *I*^2^ (%)	*p*-value for within-subgroup heterogeneity
General populations	11	2.34	(1.68, 3.27)	96.9	<0.001
Specific populations	21	2.94	(2.38, 3.63)	99.1	<0.001
Healthcare workers	13	2.62	(2.24, 3.06)	53.0	0.012

Test for subgroup differences: Q_between = 1.44, df = 2, *p* = 0.487. *p*-value for within-subgroup heterogeneity from Q-test. All subgroups were analyzed using the DerSimonian-Laird random-effects model.

### Impact of COVID-19 vaccination history on willingness to receive the influenza vaccination

**Figure 5:**
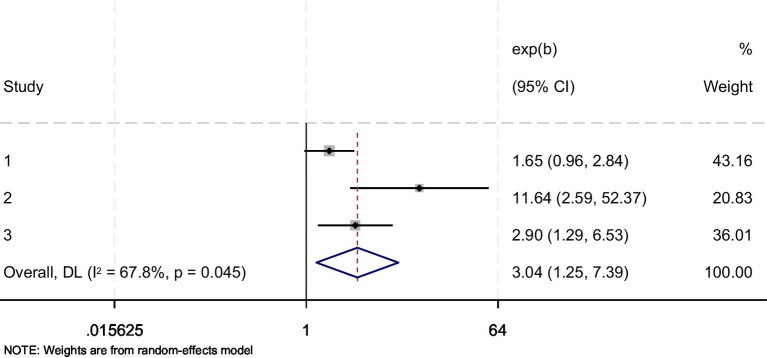
Odds ratio of COVID-19 vaccination history with influenza vaccination willingness (yes vs. no).

Three of the cross-sectional studies included in the review found that a history of COVID-19 vaccination was associated with the intention to receive the influenza vaccine (154–156). These three studies were published between 2023 and 2025 and focused on older adults, healthcare workers, and military personnel, respectively. The random-effects model showed that a history of COVID-19 vaccination was associated with a higher willingness to receive the influenza vaccine (pooled OR = 3.04, 95% CI: 1.25–7.39, *p* = 0.014); see Figure 5 for the forest plot. There was moderate heterogeneity among the three studies (*I*^2^ = 67.8%, *p* = 0.045). Given the small number of studies included in this analysis, confidence intervals were not calculated, and neither subgroup analysis nor publication bias tests were conducted. This result should be interpreted with caution, and further research is needed to validate these findings.

### Impact of COVID-19 vaccination history on influenza vaccination behavior

**Figure 6:**
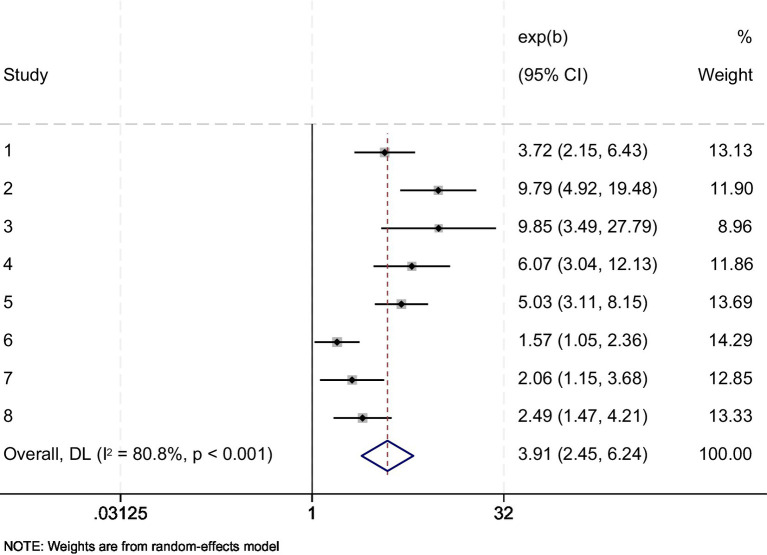
Odds ratio of COVID-19 vaccination history with influenza vaccination behavior (yes vs. no).

Eight of the cross-sectional studies included in the review found an association between a history of COVID-19 vaccination and influenza vaccination behavior (25, 157–163). These studies were published between 2021 and 2025, with sample sizes ranging from 340 to 1,261. The study populations included the general public, healthcare workers, and specific groups (older adults, pregnant women, and veterans). A meta-analysis of random-effects models showed that a history of COVID-19 vaccination was associated with higher influenza vaccination rates in the population (OR = 3.91, 95% CI: 2.45–6.24), see Figure 6 for the forest plot. There was a high degree of heterogeneity among the studies (*I*^2^ = 80.8%, *p* < 0.001), with a 95% confidence interval for the pooled odds ratio ranging from 0.81 to 18.81, representing the average association across different settings.

**Figure 7:**
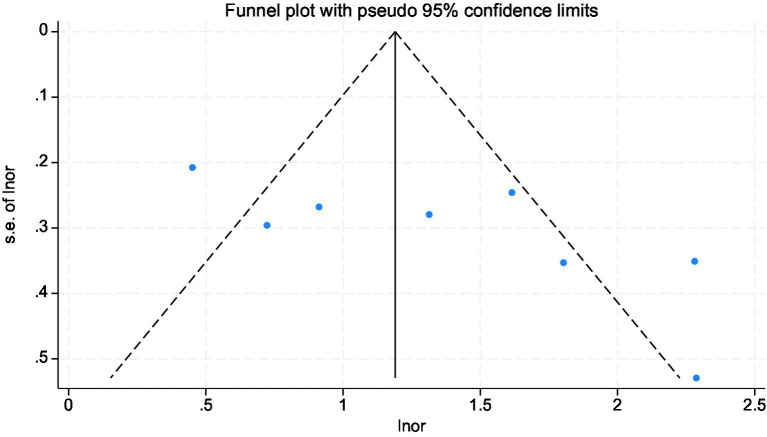
Funnel plot of the included studies (influence of COVID-19 vaccination history on influenza vaccination behavior).

Subgroup analyses were conducted to explore sources of heterogeneity. Given the scattered distribution of the 8 studies across different countries—with most countries represented by only one study—no subgroup analysis by country was performed. The results of the subgroup analysis by study population are shown in Table 6. Heterogeneity decreased in both the general population and special subgroups. Moreover, the Q-test result was significant, indicating that the study population may be a contributing factor to the high heterogeneity observed across the included studies. As shown in Figure 7, the funnel plot is relatively symmetrical, indicating minimal publication bias.

**Table 6:** Subgroup analysis by study populations (COVID-19 vaccination history and influenza vaccination behavior).

Subgroup	Number of studies	Pooled OR	95% CI	Heterogeneity *I*^2^ (%)	*p*-value for within-subgroup heterogeneity
General populations	3	6.66	(3.26, 13.64)	65.2	0.057
Specific populations	3	3.54	(1.45, 8.65)	89.0	<0.001
Healthcare workers	2	2.29	(1.55, 3.37)	0	0.635

Test for subgroup differences: Q_between = 6.77, df = 2, *p* = 0.034. *p*-value for within-subgroup heterogeneity from Q-test. All subgroups were analyzed using the DerSimonian-Laird random-effects model.

## Discussion

### Public health implications

Hesitancy and willingness to be vaccinated are more important public health measures than vaccine availability or access (164). This study aims to examine the impact of prior vaccination history on future vaccination intentions and behavior. A history of influenza vaccination is associated with a higher likelihood of receiving the COVID-19 vaccine; furthermore, a history of COVID-19 vaccination is also associated with a higher likelihood of receiving the influenza vaccine.

Several previous studies revealed a history of influenza vaccination was associated with higher odds of receiving the COVID-19 vaccine (165–168), possibly because people who have received influenza vaccines have a greater understanding of the role of vaccines in preventing respiratory diseases (169). More importantly, people who choose to receive the influenza vaccine often trust the safety and efficacy of the vaccine (170), and people with positive attitudes toward its safety and efficacy are also more receptive to other vaccines, such as the COVID-19 vaccine (128). Willingness to vaccinate is a reflection of attitudes, indicating greater trust in the vaccine and greater acceptance of other vaccines; this finding has been validated by studies of the relationship between seasonal influenza vaccination history and willingness to receive the H1N1 vaccine (171, 172).

The emergence of COVID-19 in 2019 dramatically changed the public’s perception of health risks, with a significant change in the public’s perception of the influenza vaccine and an increase in enthusiasm for vaccination (161, 173). Since the beginning of the COVID-19 pandemic, countries have been actively engaging in vaccine development and widespread vaccination to prevent infections and protect public health. The COVID-19 vaccine is effective in preventing infections; reducing disease severity, hospitalization, and mortality rates following infection (32, 174); and decreasing the occurrence of long COVID (175). The COVID-19 pandemic has led to a greater understanding of the role of vaccines in preventing viral infections. The greater willingness to receive influenza vaccines among those who had received the COVID-19 vaccine could be because those who had been vaccinated had a higher level of trust in the vaccine information released by governmental agencies and health care institutions (21, 128), were better informed about the disease, and had better vaccine cognition and personal health concepts.

People adopt healthy behaviors as a result of lifelong social learning, which begins early in life (176). By engaging in various preventive behaviors, such as vaccination, individuals develop a predisposition to health-promoting behaviors, which may make individuals more willing to be vaccinated or enable them to proactively seek accurate information about vaccines, better understand the benefits and safety of vaccines, and have more faith in vaccine efficacy later in life (177). Additionally, people with a history of vaccination may be more conscious of their health, as the more habitual they are, the less likely they are to embrace clinical preventive behaviors (178). When other vaccinations are needed, the willingness of this population to be vaccinated increases because of their health needs.

The success of a vaccination program depends on how people perceive and accept the vaccine (179). A willingness to vaccinate implies trust in government agencies (180), and having been previously vaccinated is a further behavioral signal indicating this. These individuals are more likely to follow vaccine recommendations from government agencies and health care providers, have a greater perceived need for vaccines, and have more accurate perceptions of vaccine safety and efficacy (171). Prior vaccination experience suggests increased recognition and acceptance of vaccines, resulting in positive attitudes that can contribute to the subsequent administration of other vaccines (181).

### Heterogeneity

In the meta-analysis, heterogeneity may exist if the sample estimates for the population risk were of different magnitudes (182). The *I*^2^ statistic implies the percentage of variation across selected studies that is due to heterogeneity rather than chance (183). There is a high degree of heterogeneity among the included studies, whether regarding the impact of prior influenza vaccination on COVID-19 vaccination or the impact of prior COVID-19 vaccination on influenza vaccination. Although subgroup analysis and meta-regression helped reduce heterogeneity in some subgroups, the overall level of heterogeneity remained high. These heterogeneities may reflect significant differences in the study populations (general population, healthcare workers, pregnant women, patients with chronic diseases, older adults, etc.), geographic settings (covering regions such as North America, Europe, and Asia), study periods (prior to vaccine availability, during active vaccine promotion, etc.), and research methodologies (cross-sectional surveys, registry-based cohort studies). Therefore, when considering the relationship between the two vaccinations, policymakers should take the actual circumstances fully into account. The wide confidence interval also underscores this point: although the pooled odds ratio (OR) for the association between a history of influenza vaccination and willingness to receive the COVID-19 vaccine was 2.73, its 95% confidence interval ranged from 0.66 to 11.07. This suggests that the impact of promoting influenza vaccination on future willingness to receive the COVID-19 vaccine varies within a certain range depending on the context.

### Public health practice

Previous vaccination history can help shape positive attitudes toward vaccines among populations, which is beneficial for future vaccination efforts (184). Therefore, when promoting vaccines, it is more effective to start with populations that have a history of vaccination, as this can help increase vaccination rates. This study found that there is an interaction between influenza and COVID-19 vaccination history and vaccination willingness and behavior, suggesting that increasing vaccination rates for routine vaccines (such as seasonal influenza vaccines) may make it easier for the public to accept vaccines promoted during public health emergencies (such as COVID-19 vaccines) (21).

In the post-pandemic era, global health systems face a dual challenge: on the one hand, they must restructure care pathways to address evolving health needs and limited resources; on the other hand, they must cope with the ongoing demand for rehabilitation services resulting from the long-term aftereffects of COVID-19 (185, 186). When resources are limited, if a single intervention can advance multiple public health initiatives simultaneously, it will greatly improve the efficiency of resource utilization (187). Therefore, policymakers should identify opportunities for coordinated promotion and design targeted strategies for joint vaccination, shared outreach channels, and resource allocation. For example, combining information dissemination strategies for influenza and COVID-19 may enhance public acceptance of vaccines, thereby increasing vaccination rates (24). In an environment where resources are limited but demand is increasing, this collaborative strategy can yield efficiency gains and significantly improve resource utilization.

### Limitations

The limitations of this study are as follows. (a) Most of the included studies were cross-sectional surveys, and it was not possible to determine a causal relationship between past vaccination history and willingness to receive vaccines in the future on the basis of the available information. The policy value of this meta-analysis should be focused on identifying patterns of behavioral clustering to guide coordinated interventions, rather than providing causal effects that can be directly generalized. (b) The included results may be potentially subject to error, and it is not possible to determine whether recall error or self-reporting bias existed during data collection in the original literature. (c) The study included research conducted in the United States, China, Egypt, Thailand, the United Arab Emirates, and other countries. Therefore, the generalizability of the study results may be limited outside the main countries studied. Future studies in other regions or cultural contexts may yield more valuable findings. (d) We may have missed some unpublished literature. (e) Only three studies examined the impact of COVID-19 vaccination history on influenza vaccination willingness, so no publication bias test was conducted. (f) Because vaccination rates are a common outcome and often exceed 10%, the ORs used for our outcome metrics may have overstated risk differences to some extent, and the actual rates may be lower. Risk ratios (RRs) might have been an alternative if raw numbers were available from all studies, but ORs are standard from many published observational studies. (g) Due to the limited raw data provided in individual documents, it is too difficult to calculate the pooled prevalence.

## Conclusion

This study indicates that a history of influenza vaccination and COVID-19 vaccination influence one another; specifically, a history of vaccination is associated with an increased likelihood of future vaccination. This study further underscores the importance of public perception of vaccines and provides guidance for the development of future vaccination promotion strategies.

## Data Availability

The original contributions presented in the study are included in the article/Supplementary material, further inquiries can be directed to the corresponding authors.
