# Supplementary figures and images for: Bidirectional associations between influenza and COVID-19 vaccination: a systematic review and meta-analysis
Source: Front Public Health. 2026 Jun 17;14:1756985. doi: 10.3389/fpubh.2026.1756985 (PMC13319041; doi:10.3389/fpubh.2026.1756985)

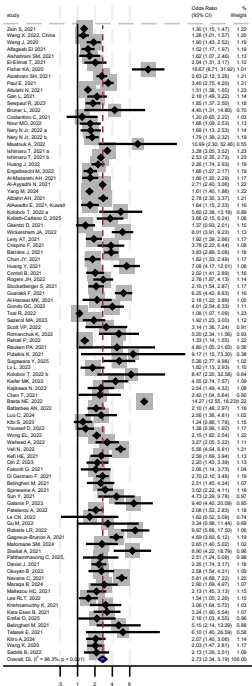

Supplement: Supplementary file 1 [file Data_Sheet_1.pdf]
