# Supplementary material for: Bidirectional associations between influenza and COVID-19 vaccination: a systematic review and meta-analysis
Source: Front Public Health. 2026 Jun 17;14:1756985. doi: 10.3389/fpubh.2026.1756985 (PMC13319041; doi:10.3389/fpubh.2026.1756985)
Supplement: Supplementary file 4 [file Table_3.DOCX]

Characteristics of the included studies.

| Author, year, country/region | Study design | Sample size | Participants | Age (years) | Adjusted confounders | Comparison | Adjusted OR (95% CI) |
| --- | --- | --- | --- | --- | --- | --- | --- |
| Zein S, 2021,Jordan, West Bank, and Syria | cross-sectional survey | 8619 | General Adults | ≥18 | Sex, Age, Nationality, Education, occupation, Employment status, Marital status, Family size | Influenza vaccination history in the last year (yes vs. no) | 1.3(1.1, 1.4) |
| Wang X, 2022, China | cross-sectional survey | 2047 | General Adults | ≥18 | Age, sex, non-healthcare staff, education, income, residence, whether herd immunity works, whether vaccination help reduce anxiety | Influenza vaccination history (yes vs. no) | 1.26(1.01, 1.57) |
| Wang J, 2020, China | cross-sectional survey | 2058 | General Adults | ≥18 | Age, sex, education, marriage status, location, region, employment status, health status, family income, there are confirmed or suspected cases in the county, perceived risk of infection, pandemic impact on daily life /work/income | Influenza vaccination history in the past season (yes vs. no) | 1.90(1.43, 2.51) |
| Alfageeh EI 2021, Saudi Arabia | cross-sectional survey | 2319 | General Adults | ≥18 | Age, sex, marital status, education, employment status, region of residence, monthly income, chronic conditions, refused vaccination in the past, contracted COVID-19, family member contracted COVID-19 | Influenza vaccination history (yes vs. no) | 1.52(1.17, 1.97) |
| Alshahrani SM, 2021, Saudi Arabia | cross-sectional survey | 758 | General Adults | ≥18 | Age, sex, education, health practitioner, chronic diseases, where do you get COVID 19 information from, knowledge of COVID 19 precautions, think the vaccine work for the new COVID 19 variants, COVID-19 previous infection, take the vaccine if it is required to travel outside KSA | Influenza vaccination history (yes vs. no) | 1.62(1.07, 2.47) |
| El-Elimat T, 2021, Jordan | cross-sectional survey | 3100 | General Adults | ≥18 | Age, sex, employment, vaccines are safe, concerned that COVID-19 pandemic is a conspiracy, not trust any information, willingness to pay for COVID-19 vaccines | Influenza vaccination history (yes vs. no) | 2.036(1.306, 3.174) |
| Fisher KA, 2020, US | cross-sectional survey | 991 | General Adults | mean age was 48 years | Age, race, sex, education, setting, perceived risk of COVID-19 (6 months) | Influenza vaccination history in the past year (no vs. yes) | 16.67(9.09, 33.33) |
| Paul E, 2021, UK | cross-sectional survey | 32361 | General Adults | ≥18 | Age, sex, education, income, employed, living arrangement, keyworker, health condition, confidence in government/health system, knowledge of COVID-19, have had COVID-19, Coronavirus anxiety scale symptoms, mistrust of vaccine benefits, worries about vaccine effects | Influenza vaccination history in the last year (no vs. yes) | 3.40(2.75, 4.20) |
| Altulahi N, 2021, Saudi Arabia | cross-sectional survey | 8056 | General Adults | ≥18 | Sex, comorbidity, allergy, smoking, age, education | Influenza vaccination history (yes vs. no) | 1.508(1.378, 1.649) |
| Gan L, 2021, China | cross-sectional survey | 1009 | General Adults | ≥18 | Age, education, trust the effectiveness of the vaccine, pay attention to the latest news of the vaccine | Influenza vaccination history (yes vs. no) | 2.176(1.474, 3.211) |
| Sewpaul R, 2023, South African | cross-sectional survey | 7962 | General Adults | ≥18 | Sex, age, population, province, community type, education, employment status, ever refused to take any vaccine | Influenza vaccination history (yes vs. no) | 1.85(1.37, 2.5) |
| Bruner L, 2022, Greece | cross-sectional survey | 55 | Adults who tested negative for COVID-19 | ≥18 | Higher frequency of worry about impact of COVID-19 on health | Influenza vaccination history in the last year (yes vs. no) | 4.4(1.3, 14.7) |
| Costantino C, 2021, Italy | cross-sectional survey | 363 | costumers of community pharmacies | Mean age was 43.9 years | Age, sex, education, influenza vaccination acceptance due to COVID pandemic | Influenza vaccination history in 2020/2021 (yes vs. no) | 1.20(0.65, 2.22) |
| Nour MO, 2022, Saudi Arabia | cross-sectional survey | 507 | General Adults | mean age of 45.2 years | Age, sex, education, family income, history of COVID-19 infection, knowledge score | Influenza vaccination history (yes vs. no) | 1.66 (1.09, 2.53) |
| Nery N Jr, 2022, Brazil | cross-sectional survey | 2521 | General Adults | ≥18 | Currently working, comorbidities, years of formal education, COVID-19 risk perception | Influenza vaccination history in 2020 (yes vs. no) | Male:1.69(1.14, 2.56); Female:1.79(1.39, 2.33) |
| Maatouk A, 2022, Tunisian | cross-sectional survey | 169 | General Adults | mean age was 48.3 years | Perceived personal risk of being infected by the coronavirus, age | Seasonal influenza vaccination history (yes vs. no) | 10.99(2.309, 52.63) |
| Ishimaru T, 2021, Japan | cross-sectional survey | 27036 | working-age population | 20-65 | Age, education, marital status, household income, underlying disease, smoking, fear of COVID-19 transmission | Influenza vaccination history during the current season (yes vs. no) | Men: 3.28(3.05, 3.52); Women:2.53 (2.35, 2.73) |
| Huang J, 2022, Global | cross-sectional survey | 2459 | General Adults | mean age was 29.31 years | Age, sex, race, education, residence, living status, work/study status, health insurance, welfare benefits | Influenza vaccination history (yes vs. no) | 2.26 (1.74, 2.93) |
| Engelbrecht M, 2022, South Africa | cross-sectional survey | 10466 | General Adults | average age was 34.5 years | Age, sex, race, education, employment, risk for COVID-19, government’s ability to roll out vaccines | Influenza vaccination history (no vs. yes) | 1.663 (1.298, 2.219) |
| Al-Mistarehi AH, 2021, Jordan | cross-sectional survey | 2208 | General Adults | mean age was 33.2 years | Sex, residence area, employment status, working in medical fields, history of COVID-19 infection, COVID-19 risk perception, perceived benefits of the vaccine, perceived clinical barriers to vaccine | Influenza vaccination history during the past 8 months (yes vs. no) | 1.655 (1.201, 2.281) |
| Yang M, 2024, China | cross-sectional study | 4,966 | General Adults | ≥ 18 | Sex; age; marriage; occupation; family members; chronic disease; COVID-19 vaccination; adverse event following immunization; COVID-19 infection; risk perception; confidence in vaccine | Influenza vaccination history in the past three years (yes vs. no) | 1.61 (1.39, 1.85) |
| Albahri AH, 2021, United Arab Emirates | cross-sectional study | 2,705 | General Adults | ≥ 18 | Age; sex; nationality; current residence; education; work status; number of adults in the family; Situation of children in the family; self-perception of health status; vaccination experience and perception; COVID-19 experience and perception | Influenza vaccination history in the past 2 years (yes vs. no) | 2.78 (2.27, 3.33) |
| AlAwadhi E, 2021, Kuwait | cross-sectional study | 7,241 | General Adults | ≥ 18 | Age; sex; marital status; trust in media; trust in doctors; trust in hospitals; probability of getting infected with influenza | Influenza vaccination history in 2019 (yes vs. no) | 1.64 (1.15, 2.33) |
| Kolobov T, 2022, Israel | cross-sectional survey | General population:503; People with diabetes:304 | adults with diabetes and general population | ≥18 | Age, sex, apprehension level of developing COVID-19, perceived rate of Israelis willing to get vaccinated against COVID-19 | Seasonal influenza vaccination history in 2020 (yes vs. no) | General population: 5.60(2.38, 13.18) |
| Al-Ayyadhi N, 2021, Kuwait | cross-sectional survey | 7274 | General Adults | ≥18 | Age, sex, edication, occupation, nationality, governorates, family member or a friend had COVID-19 infection | Influenza vaccination history (yes vs. no) | 2.706 (2.396, 3.056) |
| Alzahrani SH, 2021, Saudi Arabia | cross-sectional survey | 3048 | General Adults | ≥18 | Sex, nationality, education, geographic location, personal risk of COVID-19, risk of COVID-19 to others | Influenza vaccination history (no vs. yes) | 2.63(2.12, 3.25) |
| Kollath-Cattano C, 2025, US | cross-sectional survey | 1171 | college students | ≥18 | Sex, class standing, race, sexual orientation, parental education, employed full or part time, political ideology, reported fair or poor health, reported having COVID-19, COVID susceptibility | Influenza vaccination history in 2019-2020 (yes vs. no) | 3.66(2.15, 6.24) |
| Gkentzi D, 2021, Greece | cross-sectional survey | 399 | school teachers | 22-67 | Sex, years at work, marital status, having children, education, belief that COVID-19 vaccine should be mandatory, belief that school teachers are high risk of contracting COVID-19 | Influenza vaccination history (yes vs. no) | 1.365 (1.334, 2.864) |
| Wickersham JA, 2022, United States | cross-sectional survey | 1030 | People With HIV | ≥18 | Age, sex, race, education, annual income, sexual orientation, political orientation, median time living with HIV, CD4 >200 cells, HIV Viral Load Undetectable | Receive annual flu vaccine (yes vs. no) | 6.01(3.91, 9.22) |
| Levy AT, 2021, United States | cross-sectional survey | 662 | pregnant women | ≥18 | Age, race, location of birth, education, employment status, COVID-19 history and perceived severity, fear of COVID-19 in pregnancy, attitudes toward vaccines in general, trust the safety of vaccines | Influenza vaccination history (no vs. yes) | 1.92(1.41, 2.70) |
| Crispino F, 2021,Italy | cross-sectional survey | 276 | patients with inflammatory bowel disease | ≥18 | Household member aged, gastroenterologist advice | Influenza vaccination history(yes vs. no) | 3.78(2.22, 6.44) |
| Barrière J, 2021, French | cross-sectional survey | 999 | Cancer Patients | median age was 67 years | Age, sex | Influenza vaccination history(yes vs. no) | 3.83(2.89, 5.09) |
| Chun JY, 2021, Korea | cross-sectional survey | 1001 | Cancer Patients | mean age was 57.4 years | Age, BMI, recurrence, residence, time from initial cancer diagnosis, VAS | Influenza vaccination history within 3years (yes vs. no) | 1.82(1.33, 2.48) |
| Huang Y, 2021, UK | cross-sectional survey | 3191 | people with multiple sclerosis | ≥18 | Age, sex, education, on DMT, progressive, anxiety, motor disability, adolescent vaccines | Influenza vaccination history(yes vs. no) | 7.08(4.14, 11.92) |
| Contoli B, 2021, Italy | cross-sectional survey | 1876 | Elderly | ≥65 | Age, sex, education, economic difficulties, population density, geographic area of residence, disabilities, frailty, isolation, chronic diseases, COVID-19 attitudes and experiences | Seasonal influenza vaccination history(no vs. yes) | 2.02(1.41, 2.89); |
| Rogers JH, 2022, US | cross-sectional survey | 969(672 residents + 297 Staff) | homeless shelter residents and staff | Median age of residents was 41 years; median age of staff was 33 years | Age, race, ethnicity, sex, participant type, education, employed, underlying medical conditions, enrollments per unique participant, prior SARS-CoV-2 positive test | Influenza vaccination history(yes vs. no) | 2.78(1.89, 4.17) |
| Stuckelberger S, 2021, Switzerland | cross-sectional survey | 2064 | Pregnant and Breastfeeding Women | median age was 33 years | Age, education, professionally active, primary language, any maternal co-morbidity, impact of the SARS-COV-2 pandemic, vaccination habits and beliefs, pregnancy-related variables | Influenza vaccination history in the last year (yes vs. no) | 2.1(1.5, 2.8) |
| Guaraldi F, 2021, Italy | cross-sectional survey | 1176 | Type 2 Diabetic Patients | ≥18 | Age, sex, adverse events to previous vaccinations, education | Influenza vaccination history in 2020-2021 (yes vs. no) | 6.25(4.55, 9.09) |
| Al-Hanawi MK, 2021, Saudi Arabia | cross-sectional survey | 521 | adults with chronic diseases | ≥18 | Age, sex, marital  status, education, employment  status, location, monthly income, healthcare worker, refused vaccination in the past, family infected by COVID-19, friends infected by COVID-19, Lost family members or  friends due to complications from COVID-19 | Influenza vaccination history(no vs. yes) | 2.179(1.222, 3.888) |
| Gondo GC, 2022, US | cross-sectional survey | 1405 | Individuals living with psoriatic disease | mean age was 54.5 years | Hispanic, caucasian race, sex, age, income, biologic user, has PsA, comorbidities high risk for severe COVID-19, PsA acceptable symptom state | Influenza vaccination history in the past 12 months (yes vs. no) | 4.01(2.54, 6.33) |
| Tsai R, 2022, US | cross-sectional survey | 21294 | Individuals With Cancer, Autoimmune Diseases, or Other Serious Comorbid Conditions | median age range of 40-49 years | Trust in responsible development of COVID vaccine, prior COVID infection, political leaning, residence, age, education, smoking status, sex, cancer diagnosis, mask wearing | Influenza vaccination history(yes vs. no) | 1.08 (1.07, 1.08) |
| Sezerol MA, 2023, Turkey | cross-sectional survey | 1075 | Older Adults | 65-75 | Which COVID-19 vaccine was received before, side effects, lack of time, redundancy, ineffectiveness, forget/neglect, education | Influenza vaccination history(yes vs. no) | 1.917(1.226, 2.998) |
| Scott VP, 2022, Southern California | cross-sectional survey | 486 | low-income, Latino population receiving aid from the Supplemental Nutrition Assistance Program (SNAP) | median age was 40 years | Age, language preference, any children aged, lifetime household health condition diagnoses, any social media platforms used, never/rarely reading/talking about COVID-19, COVID-19 affect mean score | Influenza vaccination history in 2019-2020 (no vs. yes) | 3.14（1.36, 7.23） |
| Romanchuk K, 2022, Canada | cross-sectional survey | 240 | people incarcerated in 3 Canadian federal prisons | median age was 46 years | Age, ethnicity, chronic health conditions, security level | Influenza vaccination history in 2019-2020 (yes vs. no) | 5.20 (2.43, 12.00) |
| Rehati P, 2022, China | cross-sectional survey | 9153 | Adolescents | 12-17.5 | Sex, daily living, stage of schooling, region, afraid of SARS-CoV-2 transmission, potential risk of being infected with SARS-CoV-2 | Influenza vaccination history (no vs. yes) | 1.33 (1.14, 1.55) |
| Reuken PA, 2021, Germany | cross-sectional survey | 578 | liver transplant recipients and candidates | median agewas 63 years in transplant recipients and 59 years in transplant candi dates | Education, employmentstatus, media coverage on SARS-CoV-2 vaccination, immunosuppression | Influenza vaccination history in 2019-2020 or 2020-2021 (yes vs. no) | 4.80 (1.05, 21.92) |
| Puteikis K, 2021, Lithuania | cross-sectional survey | 111 | People with epilepsy | ≥18 | Vaccines are safe, vaccines may cause the infectious disease they target, vaccination is the only way to gain immunity apart from acquiring the disease itself, vaccination is useful, there is a natural decrease of viral infections, regardless of the use of vaccines, COVID-19 infection | Influenza vaccination history in 2020 (yes vs. no) | 9.17 (1.15, 73.47) |
| Sugawara Y, 2025, Japan | cross-sectional survey | 1086 | hemodialysis patients | mean age was 68.0 years | Age, sex, history of anaphylaxis, history of drug allergy, marital status, housemate >65 years old, unvaccinated housemate, information source | Influenza vaccination history (yes vs. no) | 5.26(2.78, 10.0) |
| Lv L, 2023, China | cross-sectional survey | 494 | older individuals in nursing homes and those in the community | ≥60 | Sex, COVID-19 infection risk perception, perceived safety of vaccines, chronic disease, keep an eye on the COVID-19 vaccine news, the level of the role of medical staff in vaccine recommendations | Influenza vaccination history (no vs.yes) | 1.823 (1.133–2.933) |
| Kolobov T, 2022, Israel | cross-sectional survey | General population:503; People with diabetes:304 | adults with diabetes and general population | ≥18 | Age, sex, apprehension level of developing COVID-19, perceived rate of Israelis willing to get vaccinated against COVID-19 | Seasonal influenza vaccination history in 2020 (yes vs. no) | General population: 5.60(2.38, 13.18); People with diabetes:8.47(2.20, 32.56) |
| Kiefer MK, 2022, US | cross-sectional survey | 456 | pregnant and postpartum individuals | / | Prior Covid- 19 infection, prior household COVID- 19 exposure, friend or family member has received  COVID- 19 vaccine, concerned about contracting, benefit of vaccination | Influenza vaccination history over the last year (yes vs. no) | 4.55(2.78,, 7.69) |
| Kajikawa N, 2022, Japan | cross-sectional survey | 717 | primary care patients | median age was 67.0 years | Age, sex, current smoker, has family doctor, self-rated health, recommendation by others, get information from newspaper, perceived susceptibility, perceived severity, sufficient information | Influenza vaccination history in 2020-2021 (yes vs. no) | 2.54(1.49, 4.32) |
| Chen T, 2021, China | cross-sectional survey | 813 | solid organ transplant recipients | ≥18 | Liver transplantation, main source of information on COVID-19 vaccines, education, positive intention toward influenza vaccination during the current season, perceived the importance of vaccine | Influenza vaccination history during the last season (yes vs. no) | 2.42 (1.04–5.65) |
| Basta NE, 2022, Canada | Longitudinal Study | 23819 | Adults aged 50 years or older | 50-96 | Belief in prior SARS-CoV-2 infection, consequences of COVID-19 | Influenza vaccination history in 2020 (yes vs. no) | 14.27(12.55, 16.23) |
| Battarbee AN, 2022, US | cross-sectional survey | 915 | pregnant women | ≥18 | Race, education, | Influenza vaccination history in last year (yes vs. no) | 2.1(1.5–3.0) |
| Luo C, 2024, China | cross-sectional survey | 365 | Dialysis patients | median age was 58.5 years | Health behavior; age; education; dialysis duration | Influenza vaccination history (yes vs. no) | 2.56 (1.35, 4.76) |
| Kibi S, 2023, Italy | cross-sectional survey | 5313 | Young Italians | 11-30 | Sex, age, macro region, have you been vaccinated in the past, knowledge score | Influenza vaccination history in 2020 (yes vs. no) | 1.24(0.86, 1.78) |
| Youssef D, 2022, Lebanese | cross-sectional survey | 1800 | healthcare workers | ≥18 | Sex, urbanicity, frontline workers, previously diagnosed with COVID-19, perceived barriers, Benefits of vaccine, Cues of action | Influenza vaccination history (yes vs. no) | 1.38(0.99,1.92) |
| Wong EL, 2022, Southeast Asian | cross-sectional survey | 3396 | healthcare workers | ≥18 | Region, type of health care worker, age, sex, Employment, Education | Influenza vaccination history in the last year (yes vs. no) | 2.15(1.82, 2.54) |
| Waheed A, 2022, Egypt | cross-sectional survey | 500 | healthcare workers | mean age was 33.9 ± 7.9 years | Sex, education, Having children, think SARS-CoV-2 is natural, anxious about infection, trust the healthcare facility that you work in | Influenza vaccination history (yes vs. no) | 3.268(2.048, 5.217) |
| Veli N, 2022, UK | cross-sectional survey | 5454 | healthcare workers | average agewas 46 years | Age, sex, ethnicity, occupation, index of multiple deprivation quintile, previous COVID-19, trust in employing organisation, pro-vaccine score, COVID-19 conspiracies score, Personal risk of being hospitalised with COVID-19 in the next 6 months | Influenza vaccination history over the past two seasons (yes vs. no) | 5.56(4.76, 7.14) |
| Kefi HE, 2021, Tunisian | cross-sectional survey | 398 | healthcare workers | average age was 40.5 years | Age, sex, years of service, academic training, function, | Influenza vaccination history (yes vs. no) | 2.58(1.69, 3.94) |
| Qin Z, 2023, China | cross-sectional survey | 3180 | Nursing Students and Interns | / | Age, education, familiarity of COVID-19 vaccine, vaccination interval of the domestic COVID-19, COVID-19 epidemic is under control, confidence of vaccines, reason affecting vaccination, obtained training on vaccine | Influenza vaccination history (yes vs. no) | 2.20(1.43, 3.39) |
| Fakonti G, 2021, Cyprus | cross-sectional survey | 436 | Nurses and Midwives | median age was 34 years | Age, sex, received the vaccines recommended for health professionals, vulnerable group to whom vaccination is recommended, job role, public or Private hospital | Influenza vaccination history in the last 5 years (yes vs. no) | 2.06(1.14, 3.74) |
| Di Gennaro F, 2021, Italy | cross-sectional survey | 1723 | healthcare workers | mean age was 35.5 years | Sex, age, geografic area, occupational profile, area of work, length of service, SARS-CoV-2 infection, SARS-CoV-2 Infection in family members or close contacts, information sources on SARS-CoV 2 | Influenza vaccination history in last season (yes vs. no) | 2.70(2.08, 3.45) |
| Belingheri M, 2021, Italy | cross-sectional survey | 422 | nursing students | median age was 21 years | Sex, education | Influenza vaccination history (yes vs. no) | 2.51(1.45, 4.33) |
| Spinewine A, 2021, Belgium | cross-sectional survey | 1132 | Hospital Staff | / | Sex, age, work, personal health status, COVID-19 experience, HBM—perceived susceptibility, HBM—perceived seriousness, HBM—perceived benefits of taking action, HBM—Cues to action, knowledge about COVID-19 vaccine | Influenza vaccination history in 2020 (yes vs. no) | 3.02(2.22, 4.12) |
| Sun Y, 2021, China | cross-sectional survey | 505 | healthcare workers | 18-59 | Sex, age, occupation, education, marital status, children, living with elderly individuals, worried about experiencing COVID-19, understanding of the vaccine, effect of COVID-19, | Influenza vaccination history in 2020 (yes vs. no) | 4.730(2.285, 9.794) |
| Galanis P, 2023, Greece | cross-sectional survey | 795 | nurses | mean age was 38.5 years | Age, sex, marital status, education, chronic disease, physical health, front‐line nurses, adverse reactions and discomfort experienced about COVID‐19 vaccine, concerns about the side effects of vaccine, trust in COVID‐19 vaccination | Influenza vaccination history (no vs. yes) | 9.40 (4.40–20.09) |
| Patelarou A, 2022, Albania, Cyprus, Greece, Spain and Kosovo | cross-sectional survey | 1135 | nurses | mean agewas 38.3 years | Sex, mortality group per million population, infected with COVID‐19, knowledge about COVID‐19 vaccines, trust in government, trust in doctors, fear of COVID‐19 scale | Influenza vaccination history in 2019 and 2020 (yes vs. no) | 2.08 (1.53–2.83) |
| Le CN, 2022, Vietnam | cross-sectional survey | 911 | health professions students | mean age was 20.78 years | Nationality, majors, perceived severity, perceived barriers, manufacturers not disclosing adverse effect of vaccine, mass media appreciating effectiveness and safety of vaccines | Seasonal influenza vaccination history (yes vs. no) | 1.62(0.23, 2.27) |
| Gu M, 2022, US | cross-sectional survey | 243 | healthcare workers | ≥18 | COVID-19 information source, knowing someone who died from COVID-19, sex, age, race, education, region of residence, occupation | Influenza vaccination history (yes vs. no) | 3.34(0.98, 11.49) |
| Roberts LR, 2022, Southern California | cross-sectional survey | 869 | nurses | / | Age, sex, race, prior COVID-19 diagnosis, belief that influenza is more severe than COVID-19, perceived risk of dying from COVID-19 , COVID-19 vaccine knowledge, COVID-19 origin conspiracy statements, Conservative political views | Influenza vaccination history (yes vs. no) | 9.967(5.681, 17.485) |
| Gagneux-Brunon A, 2021, French | cross-sectional survey | 2047 | healthcare workers | / | Age, sex, professions, chronic medical conditions, fear about COVID-19, perceived individual risk, vaccine hesitancy | Influenza vaccination history (yes vs. no) | 4.69(3.59, 6.11) |
| Matomane SM, 2024, South Africa | cross-sectional survey | 462 | oral health professionals | / | 5C predictors, gender, age and Covid-19 positive tests | Influenza vaccination history (yes vs. no) | 2.65(1.40, 5.03) |
| Shallal A, 2021, US | cross-sectional survey | 513 | healthcare workers | ≥18 | Age, sex, marital status, education, profession, household income, country of birth, date of move to the United States, know someone personally who has contracted COVID-19, know someone personally who has died of COVID-19 | Influenza vaccination history in the last 5 years (no vs. yes) | 8.896 (4.315, 19.252) |
| Patthammavong C, 2025, Lao People’s Democratic Republic | cross-sectional survey | 1228 | healthcare workers | average age was 36 years | Age, sex, occupation, health facility level, years in healthcare, chronic conditions, ever examined or diagnosed a patient with influenza, ever treated a patient with a life-threatening complication due to influenza | Influenza vaccination history in the last season (yes vs. no) | 2.51(1.24, 5.09) |
| Daniel J, 2021, US | cross-sectional survey | 3347 | health system personnel | ≥18 | Age, sex, viral concern, patient interaction, higher education, poor health | Influenza vaccination history in 2019-2020 (yes vs. no) | 2.35(1.75, 3.18) |
| Okuyan B, 2022, Turkey | cross-sectional survey | 961 | pharmacists | mean age was 41.3 years | Sex, professional experience as a pharmacist, HBM scale, having had contracted COVID-19, having a pharmacy staff who had contracted COVID-19, having a chronic disease associated with COVID-19 risk | Influenza vaccination history in 2019 (yes vs. no) | 2.58 (1.54, 4.30) |
| Navarre C, 2021, French | cross-sectional survey | 1964 | hospital workers | ≥18 | Sex, age, profession | Influenza vaccination history (yes vs. no) | 5.81 (4.67–7.21) |
| Maraqa B, 2024, Palestine | cross-sectional survey | 919 | hospital workers | / | Sex, profession, work division, COVID-19 vaccination, emotional exhaustion, depersonalization, personal accomplishment, mistrust of vaccine benefits, worries over unforeseen future effects | Influenza vaccination history (yes vs. no) | 2.9 (1.7–5.0) |
| Maltezou HC, 2021, Greece | cross-sectional survey | 1591 | hospital workers | / | Sex, profession, complete vaccination against hepatitis B, vaccination against pandemic A (H1N1), belief that COVID-19 vaccination should be mandatory for HCP | Influenza vaccination history in the past year (no vs. yes) | 2.13(1.45, 3.125) |
| Lee RLT, 2022, China | cross-sectional survey | 512 | Healthcare professionals and community stakeholders | ≥18 | Age | Influenza vaccination history in the past three years (yes vs. no) | 1.537 (1.047, 2.258) |
| Krishnamurthy K, 2021, Barbados | cross-sectional survey | 343 | hospital workers | ≥18 | Age, sex, marital status, nationality, chronic illness, occupation, perceived knowledge | Influenza vaccination history in 2020 (yes vs. no) | 3.06 (1.64, 5.73) |
| Kara Esen B, 2021, Turkey | cross-sectional survey | 3937 | healthcare personnel | Vaccinated: median age was 38 years; Not Vaccinated: median age was 33 years | Age, sex, occupation, status of acquiring COVID-19, delayed a vaccination recommended by a doctor before, refused a vaccine for yourself or your child because you thought it was dangerous or ineffective | Influenza vaccination history this year (no vs. yes) | 3.24(1.90, 5.55) |
| Erefai O, 2025, Morocco | cross-sectional survey | 809 | nursing  students | mean age was  20.11 years | Perceived severity of infection, lack of information about the vaccine, pre-existing health conditions, concern over rapid vaccine development, concern about side effects | Influenza vaccination history(yes vs. no) | 2.16(1.14, 5.05) |
| Belingheri M, 2021, Italy | cross-sectional survey | 421 | dentists | ≥25 | Age, sex, influenza vaccination in previous influenza seasons, COVID-19 diagnosis, | Influenza vaccination history in 2020-2021 influenza season (yes vs. no) | 5.15(2.14, 12.39) |
| Talarek E, 2021, Warsaw | cross-sectional study | 411 | Medical Students | median age was 24 years | Professions; intention to be vaccinated against Ebola; vaccine accessibility; vaccine caused pain and fever after injection; vaccine efficacy | Influenza vaccination history (yes vs. no) | 6.1 (1.4, 26.6) |
| Kitro A,2024,Thai | cross-sectional study | 811 | Health care Workers and Vulnerable Populations | Mean age of the vulnerable group was 52.7; the mean age of health care workers was 42.2 | Weight; Chronic medical conditions; history of COVID-19 infection; Attitude toward COVID-19 disease and COVID-19 vaccination | Influenza vaccination history in the past year (yes vs. no) | 2.07 (1.40, 3.06) |
| Wang K, 2020, China | cross-sectional study | 806 | Nurses | ≥18 | Age; sex; having chronic conditions; public or private; work setting; cases or suspected cases of COVID-19 encountered; susceptibility to COVID-19 | Influenza vaccination history in 2019 (yes vs. no) | 2.03 (1.47, 2.81) |
| Saddik B, 2022, United Arab Emirates | cross-sectional study | 517 | healthcare workers | ≥18 | Age; sex; knowledge about vaccines; attitude about vaccine; reasons cited for taking COVID-19 vaccine | Influenza vaccination history (yes vs. no) | 2.13 (1.29, 3.51) |
| Zhang XR,2023,China | cross-sectional survey | 62395 | employees | 18-60 | Sex, Age, Education levels, Residence, Marital status, Health status, Position | Influenza vaccination history (no vs. yes) | 0.86(0.72, 1.02) |
| Zakar R, 2022, Punjab Pakistan | cross-sectional survey | 1325 | General Adults | ≥40 | Age, Education, Monthly family income, Region, Working status, Marital Status, Access to Media, Disease status, physical activity, health status | Influenza vaccination history (yes vs. no) | 1.88(1.41, 2.50) |
| Yang H, 2023, South Korea | cross-sectional survey | 225319 | General Adults | ≥19 | Age, sex, region, BMI group, physical activity, education, economic level, occupation, marriage status, smoking, alcohol consumption, depression, hypertension, diabetes | Influenza vaccination history over the past year (yes vs. no) | 3.617(3.536,3.700) |
| Papazachariou A, 2023, Greece | retrospective cross-sectional study | 3129 | General adults of a rural area | ≥18 | Age, sex, diabetes mellitus, heart failure, other cardiovascular disease, hypertension, dyslipidemia, pulmonary disease, gastrointestinal disease, mental illness, dementia, thyroid disease | Influenza vaccination history in 2019-2020 / 2020-2021 (yes vs. no) | 2019-2020: 1.30(1.05, 1.60); 2020-2021: 2.44(1.90, 3.13) |
| Ragi ME, 2024, Lebanon | cross-sectional survey | 2028 | Lebanese adults and all Syrian adults residing in areas of low socioeconomic status. | Median age was 47 years | Sex, presence of an older adult in the household, education, asset-based wealth index, chronic illness, healthcare coverage, believing COVID-19 is a serious infection, consider themselves susceptible to a COVID-19 infection, vaccines are safe and/or effective | Influenza vaccination history (yes vs. no) | Lebanese: 1.17 (0.72, 1.91); Syrian: 1.66 (1.11, 2.49) |
| Kim S, 2023, Korea | cross-sectional survey | 620 | General Adults | ≥18 | Age, healthstatus, chronic diseases, COVID-19 vaccination intention | Influenza vaccination history in the past three years (yes vs. no) | 2.33(1.59, 3.42) |
| Harris JN, 2023, US | cross-sectional survey | 1208 | General Adults | ≥18 | Sex, race, annual income, education, political views, region of country, age of household member, health condition, tested positive for COVID-19, view COVID-19 as a major problem in community | Influenza vaccination history (yes vs. no) | 6.16 (3.00, 12.68) |
| Lomeli A, 2023, US/Mexico border | cross-sectional survey | 4964 | General Adults | median age was 44 years | Sex; age; ethnicity; education; perceived risk of infection; faith leader trust; social media contacts trust; US government trust; health care provider trust; month of enrollment | Influenza vaccination history (yes vs. no) | 3.67 (2.97, 4.55) |
| Garza N， 2023, United States | cross-sectional survey | 4185 | individuals of color | median age was 37 years | Race; income; education; insurance; trust health advice from friends; trust health advice from family; trust health advice from health care institutions | Influenza vaccination history (yes vs. no) | 5.18 (4.24, 6.32) |
| Szewczyk M, 2026, Poland | cross-sectional survey | 425 | students | average age of 21.14 ± 1.59 years | Place of residence, age | Influenza vaccination history (no vs. yes) | 0.248 (0.141, 0.439) |
| Kecojevic A, 2021, US | cross-sectional survey | 457 | College students | median age was 22 years | Age, sex, race, class level, major, health care worker, COVID-19 history, vaccine knowledge, information consumption, and trust, trust news media | Influenza vaccination history in this season (yes vs. no) | 1.97 (1.11, 3.51) |
| Kollath-Cattano C, 2025, US | cross-sectional survey | 1171 | college students | ≥18 | Sex, class standing, race, sexual orientation, parental education, employed full or part time, political ideology, reported fair or poor health, reported having COVID-19, COVID susceptibility | Influenza vaccination history in 2019-2020 (yes vs. no) | 2.09(1.59, 2.72) |
| Wang X, 2022, China | cross-sectional survey | 987 | infertile patients | average age : 32.33 ± 4.37 years | Education, annual household income, Employed, duration of infertility, therapy | Influenza vaccination history (yes vs. no) | 0.67(0.46, 0.98) |
| Viola A, 2021, Italy | a prospective study | 523 | patients with chronic inflammatory diseases | Vaccinated(average age: 48 ± 18); Not vaccinated(average age: 49 ± 18） | Age, sex, disease activity, montreal classification CD, concomitant therapy, biologic therapy, former pneumococcus vaccination | Influenza vaccination history in 2022 (yes vs. no) | 2.071(1.210, 3.545) |
| Contoli B, 2021, Italy | cross-sectional survey | 1876 | Elderly | ≥65 | Age, sex, education, economic difficulties, population density, geographic area of residence, disabilities, frailty, isolation, chronic diseases, COVID-19 attitudes and experiences | Seasonal influenza vaccination history(no vs. yes) | **COVID-19 Vaccine Hesitancy:** 2.02(1.41, 2.89) |
| Gorgui J, 2022, Canada | prospective cohort study | 603 | pregnant people | mean age was 33.5 years | Age, education, BMI, household income, residence, employment status, knowledge of COVID-19 severity/vaccine | Influenza vaccination history in the last flu season (yes vs. no) | 4.43(2.32, 9.25) |
| Haderlein TP, 2022, US | cross-sectional survey | 3474874 | Veterans aged ≥65 years | ≥65 | Age, sex, race, care assessment needs score, asthma, end-stage renal disease, chronic pulmonary disease, diabetes, heart disease, immunocompromised, liver disease, severe obesity | Influenza vaccination history(yes vs. no) | 2.28(2.22, 2.34) |
| Shariff SZ, 2022, Canada | cohort study | 23247 | homelessness | ≥18 | Age, sex, level of urbanicity, number of chronic conditions, >=1 SARS-CoV test, >=1 outpatient visit to a general practitioner, mental health-care encounter in the previous year | Influenza vaccination history in 2019-2020 or 2020-2021 seasons (yes vs. no) | 1.25(1.23,1.28) |
| Campbell J, 2023, US | cross-sectional survey | 641 | medically and socially vulnerable people | ≥18 | Age, cancer, CVD, depression, HIV/AIDS, accurate knowledge about preventing COVID-19 infection, COVID-19-related conspiracy theories/beliefs, tested for COVID-19, income, insurance type, education, looking for employment | Influenza vaccination history(yes vs. no) | 2.149(1.336, 3.457) |
| Tatar M, 2026, US | cross-sectional survey | 18155 | Adults with respiratory diseases | ≥17 | Pneumonia Vaccination, respiratory Diseases | Influenza vaccination history(yes vs. no) | 1.82(1.39, 2.42) |
| Qureshi NS, 2024, US | retrospective observational study | 21,424 | jail population | ≥18 | Age, race, sex, self-reported ever being unhoused | Influenza vaccination history in 2019- 2023 (no vs. yes) | COVID-19 vaccine refusal: 2.4 (2.0–2.8) |
| Mergenova G, 2025, Kazakhstan | cross-sectional survey | 196 | People living with human immunodeficiency virus | mean age was 44.5years | Age, education, employment, anxiety, COVID-19 vaccination access, COVID-19 vaccine attitude | Influenza vaccination history (no vs.yes) | 15.64 (3.66, 66.89) |
| Hernández-García I, 2024, Spain | cross-sectional survey | 359 | patients with multiple sclerosis | median age was 31 years | Age, sex,, multiple sclerosis type | Influenza vaccination history in 2022-2023 (yes vs. no) | Booster Dose: 27.54 (12.56, 60.37) |
| Di Giuseppe G, 2022, Italy | cross-sectional survey | 517 | detained people | ≥19 | Received information about COVID-19 vaccination from media and newspapers, need of additional information about COVID-19 vaccine, belief that COVID-19 vaccine is safe, education, working activity in the prison, having children, age, first detention | Influenza vaccination history in 2020-2021 (yes vs. no) | 6.21(1.88, 20.52) |
| Giles ML, 2023, Australia | Cohort Study | 77719 | Pregnant women | / | Hospital type, mother born in Australia, parity, number of antenatal visits , smoking, maternal first nations status, maternal age | Influenza vaccination history during this pregnancy (yes vs. no) | 1.23(1.19, 1.28) |
| Chang YW, 2024, China | cross-sectional survey | 594 | pregnant women | mean age was 31.3 years | Maternal age, health condition, exercise during pregnancy, use of health supplements, hospital, | Influenza vaccination history in the previous 3 years (annual vaccination vs. none) | 2.707 (1.241-5.906) |
| Blanchi S, 2021, Italy and France | cross-sectional survey | 417 | patients on dialysis | median age was 69 years | Think that the COVID-19 vaccine is effective, trust scientists to manage the health crisis, age, country, concerns about the safety of COVID-19 vaccines | Influenza vaccination history this year (no vs. yes) | vaccine refusal: 2.624(1.095, 6.475) |
| Ha L, 2023, United States | retrospective cohort study | 7857 | Pregnant patients | No COVID  vaccination: 33.4 ± 5.2; COVID  vaccination: 34.7 ± 4.4 | Age; multiparity; obesity; Tdap vaccination; Tdap and influenza vaccination; private insurance; race/ethnicity | Influenza vaccination history (yes vs. no) | 2.83 (2.55, 3.14) |
| Urueña A, 2023, Argentina, Brazil, Chile, Colombia, Ecuador, Paraguay, Uruguay, Venezuela | cross-sectional survey | 6555 | / | ≥15 | Age, sex, education, healthcare worker, use of closed social networks as a source of information, using non-pharmacological preventive measures, lost a close person to COVID-19 infection, history of COVID-19 | Influenza vaccination history (yes vs. no) | 7.46(6.42, 8.68) |
| Purvis SJ, 2024, US | cross-sectional survey | 679 | members of three tribal nations living on reservations in the Great Plains | 30-49 | Age, education, employment, health insurance status, comorbidities, health care trust, vaccine access, isolate ability, vaccine encouragement | Influenza vaccination history (yes vs. no) | 3.42(1.65, 7.11) |
| Ye X, 2021, China | cross-sectional survey | 2156 | healthcare workers | ≥18 | Clinical major, age, sex, work location, personal religion, hospital level, department, health condition, knowledge of vaccine, confidences of vaccine, knowledge training of vaccine | Influenza vaccination history (yes vs. no) | 1.868(1.485-2.35) |
| Viskupič F, 2023, South Dakota Board | cross-sectional survey | 980 | nurses | average age was 49 years | Partisan self-identification, age, percent of time with patients, COVID in last 12 month, evangelical | Influenza vaccination history in last season (yes vs. no) | 5.843(2.707, 12.611) |
| Ulbrichtova R, 2021, Northern Region of Slovakia | cross-sectional survey | 1277 | healthcare and non-healthcare workers | median age was 48.3 years | Age, sex, job duration, type of worker, history of COVID-19, compulsory vaccination | Influenza vaccination history (yes vs. no) | 1.97(1.12, 3.46) |
| Marinos G, 2021, Greek | cross-sectional survey | 1993 | Members of Athens Medical Association | mean age was 52.9 years | Reliable information from Greek Public Health Authorities, fear of COVID-19 vaccine side effects, vaccines are safe | Influenza vaccination history (yes vs. no) | 2.31(1.74, 3.07) |
| Avakian I, 2022, Greek | cross-sectional survey | 1136 | Health Care Workers and Administrative Officers of Primary Health Care Centers | average age was 43.8 years | Sex, age, education, health care Profession, district of employment, years of practice, vulnerable/high risk group, live with older individuals or vulnerable/high risk individuals, contact with COVID-19 patients | Seasonal influenza vaccination history(yes vs. no) | 3.29(2.08, 5.20) |
| Galanis P, 2022, Greece | cross-sectional survey | 885 | healthcare workers | mean age was 40.9 years | Sex, age, marital status, education, children < 18 years old, profession, Clinical experience, financial status, chronic disease, COVID-19 disease, living with elderly people or vulnerable groups, severity of COVID-19 | Seasonal influenza vaccination history in 2020 (yes vs. no) | 4.25 (1.86–9.75) |
| Štěpánek L, 2021, Czech | cross-sectional survey | 3550 | Hospital Staff | average age was 43.2 years | Sex, age, level of fear of COVID-19, chronic disease, history of COVID-19, job type | Seasonal influenza vaccination history (yes vs. no) | 2.74(2.12, 3.57) |
| Hubble MW, 2022, US | cross-sectional survey | 860 | Unvaccinated emergency medical services (EMS) personnel | average age was 41.1 years | Previously diagnosed with COVID, perception of greater risk of COVID infection, positive belief in effectiveness\safety of vaccine, belief in importance to receive the COVID-19 vaccine | Influenza vaccination history in the last year’s influenza season (yes vs. no) | 2.57(1.37, 4.81) |
| Peterson CJ, 2023, US | cross-sectional survey | 251 | Nurses | ≥20 | Age, sex, years practiced, highest level of nursing degree, nursing title, social distancing is effective, masks are effective, recommend the COVID-19 vaccine to a friend | Influenza vaccination history (no vs. yes) | 0.01 (0.00, 0.11) |
| Oliver K, 2022, US | cross-sectional survey | 2109 | healthcare workers | / | Age, sex, race, ethnicity, role, vaccine confidence index | Influenza vaccination history (no vs. yes) | 0.28(0.18, 0.44) |
| George G, 2023, South Africa | cross-sectional survey | 7763 | healthcare workers | / | Tested positive for COVID, perceive yourself as a risk to your patients, perceive your patients as a risk to you, bad reaction when you were vaccinated, feel obligated to get vaccinated | Influenza vaccination history(yes vs. no) | 1.99(1.56–2.55) |
| Fotiadis K, 2021, Greece | cross-sectional survey | 1456 | healthcare workers | average age was 43.1 years | Sex, education, profession, health district of employment, years of practice, vulnerable/high risk group, live with vulnerable/high risk group, know contact with COVID-19 patientsa relative or friend who has had COVID-19, | Influenza vaccination history(yes vs. no) | 3.48(2.53–4.79) |
| Doran J, 2022, Azerbaijan | cross-sectional survey | 1575 | healthcare workers | mean age was 47 years | Age, sex, previous COVID‐19 infection, household size, health status, patient‐facing role, COVID‐19 vaccination is safe | Influenza vaccination history 2019-2020 (yes vs. no) | 2.3 (1.67, 3.2) |
| Elkhayat MR, 2021, Egypt | cross-sectional survey | 341 | healthcare workers | 18-61 | Age; sex; residence; marital status; job categories; chronic medical history; COVID-19 infection history; history of family COVID-19 infection; exposure to patients with a confirmed COVID-19 diagnosis | Influenza vaccination history (yes vs. no) | 2.83 (1.56, 5.15) |
| Liang X, 2023, China | cross-sectional survey | 440 | Older Adults | ≥65 | Perceptions on COVID-19 and its vaccine; knowledge related to seasonal influenza vaccination; views on seasonal influenza vaccination; perceived barrier; peer influence related to seasonal influenza vaccination; how many of your peers will be getting the flu vaccine? | COVID-19 vaccination history (yes vs. no) | 1.65 (0.96, 2.84) |
| Nitzan I, 2024, Israel | cross-sectional survey | 648 | Defense forces soldiers | median age was 20 years | Age, sex, vaccination for influenza during previous season, past confirmed covid-19, perceived effect of the covid-19 pandemic on willingness toward  influenza vaccine | COVID-19 Vaccination history (yes vs. no) | 11.64 (2.59, 52.42) |
| Lounis M, 2025, Algerian | cross-sectional survey | 112 | healthcare workers | ≥18 | Profession, place of work | COVID-19 Vaccination history (yes vs. no) | 2.899(1.29, 6.536) |
| Andrejko KL, 2023, California (USA) | cross-sectional survey | 1261 | general-population | median age was 35 years | Age, sex, race, reported comorbidity, region, SARS-CoV-2 Infection Status, use of face masks in indoor public settings, attended social gathering | COVID-19 Vaccination history (yes vs. no) | 3.72(2.15, 6.43) |
| Hamzat H, 2025, US | cross-sectional survey | 426 | General adults | ≥18 | Sex, age, political affiliation, education, income, employment, chronic conditions, trust in Public Health, trust in doctors in general, trust in Community Pharmacists | COVID-19 Vaccination history (yes vs. no) | 9.790(4.923, 19.467) |
| Guo M, 2023, China | cross-sectional survey | 833 | Community residents | / | Sex; education; the potential for COVID-19 infection; willing to continue to get the COVID-19 vaccine in the future; adverse reactions after COVID-19 vaccination | COVID-19 vaccination history (yes vs. no) | 9.85 (3.49, 27.78) |
| Kim B, 2023, Korea | cross-sectional survey | 351 | pregnant women | ≤44 | Age, parity, history of miscarriage, education | COVID-19 Vaccination history (yes vs. no) | 6.07(3.04, 12.13) |
| Patel UC, 2023, United States | cross-sectional survey | 980 | Veteran | received COVID-19 vaccine; 69.2 ± 11.6 not received COVID-19 vaccine: 63.7 ± 15 | Sex; age; race; ethnicity; high-risk population | COVID-19 vaccination history (yes vs. no) | 5.03 (3.15, 8.26) |
| You Y, 2023, China | cross-sectional survey | 975 | Older Adults | ≥60 | Sex; age; marriage status; education level; monthly wage; self-health status report; chronic medical conditions; received COVID-19 vaccine; knowledge of vaccine | COVID-19 vaccination history during the COVID-19 period (yes vs. no) | 1.57 (1.05, 2.37) |
| Rachiotis G, 2021, Greek | cross-sectional survey | 340 | healthcare workers | mean age was 44.7 years | Vaccinations are important for the protection of health care workers, vaccines are effective | COVID-19 Vaccination history (yes vs. no) | 2.06(1.15, 3.67) |
