# Supplementary material for: Bidirectional associations between influenza and COVID-19 vaccination: a systematic review and meta-analysis
Source: Front Public Health. 2026 Jun 17;14:1756985. doi: 10.3389/fpubh.2026.1756985 (PMC13319041; doi:10.3389/fpubh.2026.1756985)
Supplement: Supplementary file 5 [file Table_4.DOCX]

Quality evaluation results of JBI included in the study.

| Study | [1] | [2] | [3] | [4] | [5] | [6] | [7] | [8] | Overall quality |
| --- | --- | --- | --- | --- | --- | --- | --- | --- | --- |
| Wang J, 2020 | Y | Y | Y | Y | Y | Y | Y | Y | 8/8 |
| Zein S, 2021 | Y | Y | Y | Y | Y | Y | Y | Y | 8/8 |
| Wang X, 2022 | Y | Y | Y | Y | Y | Y | Y | Y | 8/8 |
| Alfageeh EI, 2021 | Y | Y | Y | Y | Y | Y | Y | Y | 8/8 |
| Alshahrani SM, 2021 | Y | Y | Y | Y | Y | Y | Y | Y | 8/8 |
| El-Elimat T, 2021 | Y | Y | Y | Y | Y | Y | Y | Y | 8/8 |
| Fisher KA, 2020 | Y | Y | Y | Y | Y | Y | Y | Y | 8/8 |
| Alzahrani SH, 2021 | Y | Y | Y | Y | Y | Y | Y | Y | 8/8 |
| Paul E, 2021 | Y | Y | Y | Y | Y | Y | Y | Y | 8/8 |
| Altulahi N, 2021 | Y | Y | Y | Y | Y | Y | Y | Y | 8/8 |
| Gan L, 2021 | Y | Y | Y | Y | Y | Y | Y | Y | 8/8 |
| Sewpaul R, 2023 | Y | Y | Y | Y | Y | Y | Y | Y | 8/8 |
| Galle F, 2021 | Y | Y | Y | Y | Y | Y | Y | Y | 8/8 |
| Costantino C, 2021 | Y | Y | Y | Y | Y | N | Y | Y | 7/8 |
| Nour MO, 2022 | Y | Y | Y | Y | Y | Y | Y | Y | 8/8 |
| Nery N, 2022 | Y | Y | Y | Y | Y | Y | Y | Y | 8/8 |
| Maatouk A, 2022 | Y | Y | Y | Y | Y | Y | Y | Y | 8/8 |
| Ishimaru T, 2021 | Y | Y | Y | Y | Y | Y | Y | Y | 8/8 |
| Huang J, 2022 | Y | Y | Y | Y | Y | Y | Y | Y | 8/8 |
| Engelbrecht M, 2022 | Y | Y | Y | Y | Y | Y | Y | Y | 8/8 |
| Al-Mistarehi AH, 2021 | Y | Y | Y | Y | Y | Y | Y | Y | 8/8 |
| Al-Ayyadhi N, 2021 | Y | Y | Y | Y | Y | Y | Y | Y | 8/8 |
| Yang M, 2024 | Y | Y | Y | Y | Y | Y | Y | Y | 8/8 |
| Albahri AH, 2021 | Y | Y | Y | Y | Y | Y | Y | Y | 8/8 |
| AlAwadhi E, 2021 | Y | Y | Y | Y | Y | Y | Y | Y | 8/8 |
| Gkentzi D, 2021 | Y | Y | Y | Y | Y | Y | Y | Y | 8/8 |
| Kolobov T, 2022 | Y | Y | Y | Y | Y | Y | Y | Y | 8/8 |
| Kollath-Cattano C, 2025 | Y | Y | Y | Y | Y | Y | Y | Y | 8/8 |
| Crispino F, 2021 | Y | Y | Y | Y | Y | Y | Y | Y | 8/8 |
| Barrière J, 2021 | Y | Y | Y | Y | Y | Y | Y | Y | 8/8 |
| Wickersham JA, 2022 | Y | Y | Y | Y | Y | Y | Y | Y | 8/8 |
| Levy AT, 2021 | Y | Y | Y | Y | Y | Y | Y | Y | 8/8 |
| Huang Y, 2021 | Y | Y | Y | Y | Y | Y | Y | Y | 8/8 |
| Contoli B, 2021 | Y | Y | Y | Y | Y | Y | Y | Y | 8/8 |
| Chun JY, 2021 | Y | Y | Y | Y | Y | Y | Y | Y | 8/8 |
| Stuckelberger S, 2021 | Y | Y | Y | Y | Y | Y | Y | Y | 8/8 |
| Guaraldi F, 2021 | Y | Y | Y | Y | Y | Y | Y | Y | 8/8 |
| Rogers JH, 2022 | Y | Y | Y | Y | Y | Y | Y | Y | 8/8 |
| Al-Hanawi MK, 2021 | Y | Y | Y | Y | Y | Y | Y | Y | 8/8 |
| Gondo GC, 2022 | Y | Y | Y | Y | Y | Y | Y | Y | 8/8 |
| Tsai R, 2022 | Y | Y | Y | Y | Y | Y | Y | Y | 8/8 |
| Scott VP, 2022 | Y | Y | Y | Y | Y | Y | Y | Y | 8/8 |
| Romanchuk K, 2022 | Y | Y | Y | Y | Y | Y | Y | Y | 8/8 |
| Rehati P, 2022 | Y | Y | Y | Y | Y | Y | Y | Y | 8/8 |
| Reuken PA, 2021 | Y | Y | Y | Y | Y | Y | Y | Y | 8/8 |
| Puteikis K, 2021 | Y | Y | Y | Y | Y | Y | Y | Y | 8/8 |
| Sugawara Y, 2025 | Y | Y | Y | Y | Y | Y | Y | Y | 8/8 |
| Lv L, 2023 | Y | Y | Y | Y | Y | Y | Y | Y | 8/8 |
| Kolobov T, 2022 | Y | Y | Y | Y | Y | Y | Y | Y | 8/8 |
| Kiefer MK, 2022 | Y | Y | Y | Y | Y | Y | Y | Y | 8/8 |
| Kollath-Cattano C, 2025 | Y | Y | Y | Y | Y | Y | Y | Y | 8/8 |
| Gkentzi D, 2021 | Y | Y | Y | Y | Y | Y | Y | Y | 8/8 |
| Kibi S, 2023 | Y | Y | Y | Y | Y | Y | Y | Y | 8/8 |
| Luo C, 2024 | Y | Y | Y | Y | Y | Y | Y | Y | 8/8 |
| Youssef D, 2022 | Y | Y | Y | Y | Y | Y | Y | Y | 8/8 |
| Wong ELY, 2022 | Y | Y | Y | Y | Y | Y | Y | Y | 8/8 |
| Waheed A, 2022 | Y | Y | Y | Y | Y | Y | Y | Y | 8/8 |
| El Kefi H, 2021 | Y | Y | Y | Y | Y | Y | Y | Y | 8/8 |
| Veli N, 2022 | Y | Y | Y | Y | Y | Y | Y | Y | 8/8 |
| Fakonti G, 2021 | Y | Y | Y | Y | Y | Y | Y | Y | 8/8 |
| Di Gennaro F, 2021 | Y | Y | Y | Y | Y | Y | Y | Y | 8/8 |
| Qin Z, 2023 | Y | Y | Y | Y | Y | Y | Y | Y | 8/8 |
| Spinewine A, 2021 | Y | Y | Y | Y | Y | Y | Y | Y | 8/8 |
| Sun Y, 2021 | Y | Y | Y | Y | Y | Y | Y | Y | 8/8 |
| Gu M, 2022 | Y | Y | Y | Y | Y | Y | Y | Y | 8/8 |
| Roberts LR, 2022 | Y | Y | Y | Y | Y | Y | Y | Y | 8/8 |
| Le CN, 2022 | Y | Y | Y | Y | Y | Y | Y | Y | 8/8 |
| Patthamavong C, 2025 | Y | Y | Y | Y | Y | Y | Y | Y | 8/8 |
| Parente DJ, 2021 | Y | Y | Y | Y | Y | Y | Y | Y | 8/8 |
| Okuyan B, 2021 | Y | Y | Y | Y | Y | Y | Y | Y | 8/8 |
| Maraqa B, 2024 | Y | Y | Y | Y | Y | Y | Y | Y | 8/8 |
| Maltezou HC, 2021 | Y | Y | Y | Y | Y | Y | Y | Y | 8/8 |
| Lee RLT, 2022 | Y | Y | Y | Y | Y | Y | Y | Y | 8/8 |
| Navarre C, 2021 | Y | Y | Y | Y | Y | Y | Y | Y | 8/8 |
| Krishnamurthy K, 2021 | Y | Y | Y | Y | Y | Y | Y | Y | 8/8 |
| Kara Esen B, 2021 | Y | Y | Y | Y | Y | Y | Y | Y | 8/8 |
| Erefai O, 2025 | Y | Y | Y | Y | Y | Y | Y | Y | 8/8 |
| Butsing N, 2024 | Y | Y | Y | Y | Y | Y | Y | Y | 8/8 |
| Belingheri M, 2021 | Y | Y | Y | Y | Y | Y | Y | Y | 8/8 |
| Talarek E, 2021 | Y | Y | Y | Y | Y | Y | Y | Y | 8/8 |
| Kitro A, 2024 | Y | Y | Y | Y | Y | Y | Y | Y | 8/8 |
| Wang K, 2020 | Y | Y | Y | Y | Y | Y | Y | Y | 8/8 |
| Saddik B, 2022 | Y | Y | Y | Y | Y | Y | Y | Y | 8/8 |
| Zhang XR, 2023 | Y | Y | Y | Y | Y | Y | Y | Y | 8/8 |
| Yang H, 2023 | Y | Y | Y | Y | Y | Y | Y | Y | 8/8 |
| Zakar R, 2022 | Y | Y | Y | Y | Y | Y | Y | Y | 8/8 |
| Harris JN, 2023 | Y | Y | Y | Y | Y | Y | Y | Y | 8/8 |
| Ragi ME, 2024 | Y | Y | Y | Y | Y | Y | Y | Y | 8/8 |
| Kim S, 2023 | Y | Y | Y | Y | Y | Y | Y | Y | 8/8 |
| Kollath-Cattano C, 2025 | Y | Y | Y | Y | Y | Y | Y | Y | 8/8 |
| Kecojevic A, 2021 | Y | Y | Y | Y | Y | Y | Y | Y | 8/8 |
| Szewczyk M, 2026 | Y | Y | Y | Y | Y | Y | Y | Y | 8/8 |
| Wang X, 2022 | Y | Y | Y | Y | Y | Y | Y | Y | 8/8 |
| Haderlein TP, 2022 | Y | Y | Y | Y | Y | Y | Y | Y | 8/8 |
| Viola A, 2021 | Y | Y | Y | Y | Y | Y | Y | Y | 8/8 |
| Contoli B, 2021 | Y | Y | Y | Y | Y | Y | Y | Y | 8/8 |
| Gorgui J, 2022 | Y | Y | Y | Y | Y | Y | Y | Y | 8/8 |
| Hernandez-Garcia I, 2024 | Y | Y | Y | Y | Y | Y | Y | Y | 8/8 |
| Campbell J, 2023 | Y | Y | Y | Y | Y | Y | Y | Y | 8/8 |
| Tatar M, 2025 | Y | Y | Y | Y | Y | Y | Y | Y | 8/8 |
| Mergenova G, 2025 | Y | Y | Y | Y | Y | Y | Y | Y | 8/8 |
| Blanchi S, 2021 | Y | Y | Y | Y | Y | Y | Y | Y | 8/8 |
| Di Giuseppe G, 2022 | Y | Y | Y | Y | Y | Y | Y | Y | 8/8 |
| Chang YW, 2024 | Y | Y | Y | Y | Y | Y | Y | Y | 8/8 |
| Purvis SJ, 2024 | Y | Y | Y | Y | Y | Y | Y | Y | 8/8 |
| Urueña A, 2023 | Y | Y | Y | Y | Y | Y | Y | Y | 8/8 |
| Ulbrichova R, 2021 | Y | Y | Y | Y | Y | Y | Y | Y | 8/8 |
| Ye X, 2021 | Y | Y | Y | Y | Y | Y | Y | Y | 8/8 |
| Viskupic F, 2022 | Y | Y | Y | Y | Y | Y | Y | Y | 8/8 |
| Avakian I, 2022 | Y | Y | Y | Y | Y | Y | Y | Y | 8/8 |
| Galanis P, 2022 | Y | Y | Y | Y | Y | Y | Y | Y | 8/8 |
| Marinos G, 2021 | Y | Y | Y | Y | Y | Y | Y | Y | 8/8 |
| Peterson CJ, 2023 | Y | Y | Y | Y | Y | Y | Y | Y | 8/8 |
| Štěpánek L, 2021 | Y | Y | Y | Y | Y | Y | Y | Y | 8/8 |
| Hubble MW, 2022 | Y | Y | Y | Y | Y | Y | Y | Y | 8/8 |
| Oliver K, 2022 | Y | Y | Y | Y | Y | Y | Y | Y | 8/8 |
| Garza 2023 | Y | Y | Y | Y | Y | Y | Y | N | 7/8 |
| Lomeli 2023 | Y | Y | Y | Y | Y | Y | Y | N | 7/8 |
| Elkhayat 2021 | Y | Y | Y | Y | Y | Y | Y | Y | 8/8 |
| Ha 2023 | Y | Y | Y | Y | Y | Y | Y | Y | 8/8 |
| Lounis 2025 | Y | Y | Y | Y | Y | Y | Y | N | 7/8 |
| Nitzan 2024 | Y | Y | Y | Y | Y | Y | Y | Y | 8/8 |
| Liang 2023 | Y | Y | Y | Y | Y | Y | Y | Y | 8/8 |
| Andrejko 2023 | Y | Y | Y | Y | Y | Y | Y | Y | 8/8 |
| Hamzat 2025 | Y | Y | Y | Y | Y | Y | Y | Y | 8/8 |
| Kim 2023 | Y | Y | Y | Y | Y | Y | Y | Y | 8/8 |
| Rachiotis 2021 | Y | Y | Y | Y | Y | Y | Y | N | 7/8 |
| You 2023 | Y | Y | Y | Y | Y | Y | Y | Y | 8/8 |
| Guo 2023 | Y | Y | Y | Y | Y | Y | Y | Y | 8/8 |
| Patel 2023 | Y | Y | Y | Y | Y | Y | Y | Y | 8/8 |
| Papazachariou 2023 | Y | Y | Y | Y | Y | N | Y | N | 6/8 |
| Basta 2022 | Y | Y | Y | Y | Y | Y | Y | Y | 8/8 |
